# Comparison and clinical analysis of antibiotics and endoscopic injection for vesicoureteral reflux in children

**DOI:** 10.1007/s00383-024-05780-3

**Published:** 2024-07-12

**Authors:** Zhen Yang, Kanglin Dai, Xianglun Sun, Chen Tian, Lihua Yuan, Jingsi Liu, Ban Li, Patrick Ho Yu Chung, Kenneth Kak Yuen Wong

**Affiliations:** 1 Division of Pediatric Surgery, Department of Surgery, The University of Hong Kong Shenzhen Hospital, Shen Zhen, China; 2 Division of Pediatric Surgery, Department of Surgery, The University of Hong Kong, Queen Mary Hospital, Pokfulam Road, Hong Kong SAR, China

**Keywords:** Vesicoureteral reflux, Continuous antibiotic prophylaxis, Endoscopic injections

## Abstract

**Purpose:**

This study evaluated the outcome of pediatric patients with primary vesicoureteral reflux (VUR) and compared of the treatments between continued antibiotic prophylaxis (CAP) and endoscopic injection.

**Methods:**

The clinical data of children diagnosed with primary vesicoureteral reflux from March 2015 to June 2020 who were treated with antibiotics or endoscopic injection were reviewed. Antibiotic was the first-chosen treatment after the diagnosis of VUR in children. Endoscopic treatment consisted of injection of dextran hyaluronic acid copolymer (DX/HA) into the ureteral opening under direct cystoscopy guidance.

**Results:**

Fifty-two children (35 males, 17 females) were included in this study, and for a total 90 ureters (14 unilateral, 38 bilateral) were diagnosed with vesicoureteral reflux by Voiding cystourethrography (VCUG). Twenty-two children were treated with antibiotics (8 unilateral, 14 bilateral), for a total of 36 ureters; thirty children were treated by endoscopic injection (6 unilateral, 24 bilateral), for a total of 54 ureters. The injection surgery took 36 ± 17 min including duration of general anesthesia and circumcision and the hospital stay was 2.3 ± 1.3 days. All male patients underwent circumcision simultaneously. There were no drug and allergic reactions in the antibiotic group, and no postoperative complications occurred in the injection group. With 23 months (13–63 months) of mean follow-up, the resolution rate, defined as radiological disappearance of VUR, was 36.1% (13/36) in the antibiotic group and 57.4% (31/54) in the injection group (*P* = 0.048).Two cases of bilateral reflux in the injection group required a second injection before resolution could be achieved. Thus, the overall success rate of injection was 64.8% (35/54). 9 cases (9/18, 50%) in the antibiotic group had renal scars on DMSA scans, while this was seen in 20 cases (20/23, 86.9%) in the injection group. There was a statistically significant difference between the two groups (*P* = 0.010).The positive rates of ultrasound between the antibiotic group and the injection group were 45.5% (10/22) and 80.0% (24/30), respectively. There was a statistically significant difference between the two groups in positive rates of ultrasound (*P* = 0.010).

**Conclusions:**

Endoscopic injection is easy to operate with short surgical time and hospital stay, so it is a safe and feasible treatment. For the treatment of primary vesicoureteral reflux in children, the radiological resolution rate of endoscopic injection is better than antibiotic therapy. In this study, the presence of kidney scars on DMSA and the dilated of the collecting system on ultrasound are the indications for endoscopic injection.

## Introduction

Vesicoureteral reflux (VUR) is one of the most common congenital urinary malformations. VUR has the potential to resolve spontaneously. It is estimated that 80% of low-grade VUR will spontaneously disappear in 5 years and 30–50% in high-grade reflux [1]. Due to a high resolution rate, continued antibiotic prophylaxis (CAP) against breakthrough infection is a widely accepted treatment, while surgical treatment is reserved for persistent or complicated cases. There is no consensus on the role of Continuous antibiotic prophylaxis (CAP), but guidelines in both the United States and Europe recommend infection prevention as an initial treatment for infants under 1 year of age with high-grade reflux [2, 3]. Operative treatment for VUR can be divided into open, laparoscopic and endoscopic surgery. Open and laparoscopic surgical repair in the form of ureteric implantation has a high success rate of approximately 90–95% in elder children [4]. However, it is technically difficult in infant bladder, and postoperative complications leading to prolonged hospitalization may occur.

In 2010, the American Urological Association (AUA) guidelines incorporated endoscopic injection into the management of VUR as an alternative treatment option other than the conventional managements. Following approval by the FDA, VUR patients worldwide are increasingly receiving endoscopic injections to treat VUR. Since 2015, our center began to introduce Deflux injections as the first line of treatment for children with VUR. Meta-analyses and systematic reviews report an overall success rate of 77–85% [5–7].

Obviously, endoscopic injection for VUR has become an alternative not only to open surgery, but also to conservative treatment. Therefore, the purpose of our study was to make a comparative analysis of the two treatments for primary VUR between continuous prophylactic antibiotic therapy and endoscopic injection therapy.

## Materials and methods

This study was retrospective comparative research and collected clinical data of 58 children with vesicoureteral reflux from the University of Hong Kong Shenzhen Hospital and the University of Hong Kong Mary Hospital from March 2015 to June 2020.

### Inclusion criteria

1. Primary vesicoureteral reflux: diagnosed with voiding cystourethrography (VCUG).

2. Age: < 16 years.

### Exclusion criteria

1. Secondary VUR: Neurogenic bladder, posterior urethral valve, etc.

2. Age: > 16 years.

3. Operation history.

4. During the follow-up period, the actual treatment process and clinical features cannot be provided.

The clinical data were reviewed and summarized. According to different treatment methods, the patients in the study were divided into two groups: continuous prophylactic antibiotic group (Antibiotic group) and endoscopic injection group (Injection group).

Antibiotic was the first-chosen treatment after the diagnosis of VUR was established in children. It was used to treat any febrile urinary tract infection and continued antibiotic prophylaxis (CAP) will be initiated. For children with febrile UTI, the total course of antibiotic therapy should be 7–14 days, whether the initial route of administration of antibiotics is oral or parenteral. The usual choices for oral antibiotics include a first-generation cephalosporine or amoxicillin-clavulanic acid. Antibiotic prophylaxis is more often recommended for children with high-grade reflux. During the follow-up process, VCUG was rechecked each year after diagnosis, and confirm the grade of reflux.

Endoscopic treatment consisted of injection of dextran hyaluronic acid copolymer (DX/HA) into the ureteral opening under direct cystoscope guidance. All injection procedures were done under general anesthesia with the patient in cystolithotomy position. A single dose of intravenous antibiotics was given immediately before the operation. All male patients underwent circumcision simultaneously. A pediatric rigid cystoscope was used for the transurethral injection. The injection technique used initially was the classic STING technique. The needle was inserted subureterically 2 to 3 mm below the orifice and the tip was advanced under the distal ureter. This will re-create a submucosal tunnel to achieve an anti-reflux effect. The ureteral orifice would have a slit like appearance and the ureteral orifice would have a ‘volcano’ appearance. VCUG was performed at 12 weeks after repeated injections. Antibiotic prophylaxis was discontinued if resolution of VUR is confirmed.

This study analysis was performed with SPSS 22.0 software. Quantitative data was presented using mean ± standard deviation for normal distribution and homogeneity of variance. Independent sample *t* test was used for comparison between two groups, while median (Q25, Q75) was used for comparison. Nonparametric rank sum test was used for comparison between groups; Qualitative data is expressed in percentage (%), and intergroup comparisons are conducted using chi square test and exact probability method. In all analyses, a *p* value of < 0.05 was considered to be statistically significant.

## Results

According to the inclusion and exclusion criteria, 6 cases were excluded, including three cases of secondary vesicoureteral reflux (neurogenic bladder) and 3 cases loss to follow-up. Finally, 52 children (35 males, 17 females) were included in this study, and a total of 90 ureters (14 unilateral, 38 bilateral) were diagnosed with vesicoureteral reflux by Voiding cystourethrography examination (Table 1). Twenty-two children were treated with antibiotics (8 unilateral, 14 bilateral), for a total of 36 ureters; Thirty children were treated by endoscopic injection (6 unilateral, 24 bilateral), for a total of 54 ureters (Table 2). The injection surgery took 36 ± 17 min and the hospital stay was 2.3 ± 1.3 days. All male patients underwent circumcision simultaneously. There were no drug and allergic reactions in the antibiotic group, and no postoperative complications occurred in the injection group.

**Table 1:** Demographic data and patient characteristics

Total		*N* = 52	%
Gender	Male	35	67.3
	Female	17	32.7
Laterality	Left	12	23.07
	Right	2	3.84
	Bilateral	38	73.08
Urinary tract infection		48	92.3
Congenital malformation		8	15.38
Antenatal diagnosis		6	11.54
Ureteral Units	Grade	*N* = 90	
	I	2	2.22
	II	9	10
	III	34	37.78
	IV	30	33.33
	V	15	16.67

**Table 2:** Patient characteristics of Antibiotic group and Injection group

Group		Antibiotic		Injection		*P* value
		*N* = 22	%	*N* = 30	%	
Gender	Male	17	77.3	18	60.0	0.190
	Female	5	22.7	12	40.0	
Laterality	Left	7	31.8	5	16.7	0.411
	Right	1	4.5	1	3.3	
	Bilateral	14	63.6	24	80.0	
Grade	I-III	22	61.1	23	42.6	0.085
	IV-V	14	38.9	31	57.4	
Age	Months	6.5 (3.00,12.50)		12.50 (9.00,23.25)		0.003

**Fig. 1:**
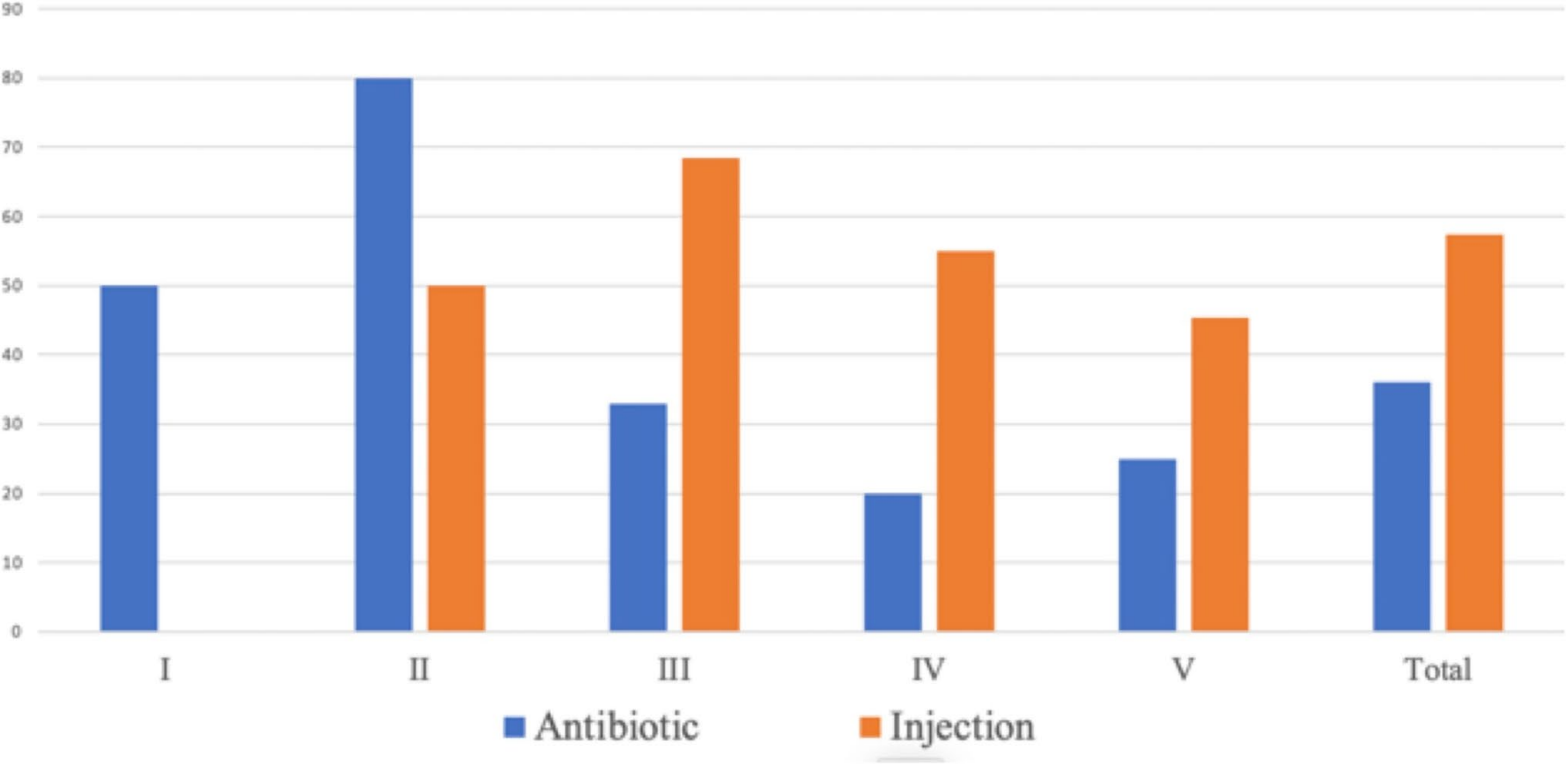
Resolution rates of VUR with antibiotic treatment versus Deflux injection

With 23 months (13–63 months) of follow-up, the resolution rate, defined as radiological disappearance of VUR, was 36.1%(13/36) in the antibiotic group and 57.4% (31/54) in the injection group (*P* = 0.048) (Table 3, Fig. 1). Two cases of bilateral reflux in the injection group required a second injection before resolution could be achieved. Thus, the overall success rate was 64.8% (35/54).Ureteral reimplantation (Cohen) was performed in 2 cases, each one in the antibiotic and injection groups, due to recurrent febrile urinary tract infections while on prophylactic antibiotics associated with new renal damage on DMSA scan.

**Table 3:** Success rate of Vesicoureteral Reflux Ureteral Units

Grade	Antibiotic(*N* = 36)			Injection(*N* = 54)			*P* value
	Resolved	Non resolved	Total	Resolved	Non resolved	Total	
I	1 (50%)	1	2				
II	4 (80%)	1	5	2 (50%)	2	4	
III	5 (33%)	10	15	13 (68.4%)	6	19	
IV	2 (20%)	8	10	11 (55%)	9	20	
V	1 (25%)	3	4	5 (45.4%)	6	11	
Total	13 (36.1%)	23	36	31 (57.4%)	23	54	0.048

In DMSA scan, 9 cases (9/18, 50%) in the antibiotic group showed renal scars, while 20 cases (20/23, 86.9%) in the injection group. There was a statistically significant difference between the two groups (*P* = 0.010).The positive rates of ultrasound between the antibiotic group and the injection group were 45.5% (10/22) and 80.0% (24/30), respectively. There was a statistically significant difference between the two groups in positive rates of ultrasound (*P* = 0.010) (Table 4).The difference of renal function < 40% was not statistically significant (*P* = 0.262).

**Table 4:** Imaging examination of Antibiotic group and Injection group

	Antibiotic(*N* = 22)		Injection(*N* = 30)		*P* value
	Positive/Total	%	Positive/Total	%	
Antenatal diagnosis	2/22	9.1	4/30	13.3	0.636
Congenital urinary malformation	4/22	18.2	4/30	13.3	0.632
Ultrasound	10/22	45.5	24/30	80.0	0.010
Renal scars (DMSA)	9/18	50.0	20/23	86.9	0.010
Split renal function < 40% (DMSA)	7/18	38.8	13/23	56.5	0.262

## Discussion

Vesicoureteral reflux is one of the most common congenital urinary malformations. The prevalence in the normal children is 0.8–1.4% [8], and 30% to 40% of children with Urinary tract infection (UTI) are diagnosed with VUR [9, 10]. VUR has the potential to resolve spontaneously. It is well known that VUR tends to resolve over time and that resolution depends mainly on patient age, gender, and grade of VUR. The resolve rate is proportional to the level of primary reflux; Approximately 80% for low-level reflux (grade I and II) will spontaneously resolve, 50% for grade III and 20% for high-level (grade IV and V) [1]. Schwab et al. have demonstrated that grade I to III reflux resolve at 13% yearly during the initial 5 years of follow-up and then at 3.5% yearly during subsequent follow-up. Grade IV to V reflux resolve at 5% rate yearly [11]. In our study, we defined resolve rate as radiological disappearance of VUR on VCUG. The resolve rates in the antibiotic group of children were 50.0% for grade I, 80.0% for grade II, 33.3% for grade III, 20.0% for grade IV and 25.0% for grade V.

Today, two possible imaging strategies have been proposed for the diagnosis of VUR in patients with UTI. In the traditional "bottom-up" approach, ultrasound examination and VCUG examination are first performed for initial diagnosis, and only DMSA renal isotope scanning is performed for patients with high-grade VUR or recurrent UTI [3]. In our study, the “bottom-up” approach for all 52 children with reflux. Among them, 41 children underwent DMSA examination, and the examination time was simultaneously or delayed with VCUG time. Among them, 29 patients (29/41, 70.7%) had abnormal results, which were manifested as focal parenchymal defects, and the deficiency on the nephogram indicated the presence of renal scar. Compared with the two groups with different treatment methods, the difference of renal scar performance was statistically significant (*P* = 0.010), but the difference of renal function < 40% was not statistically significant (*P* = 0.262). The reason why the difference in renal function < 40% between the two groups was not statistically significant may be that in our study, children with reflux had a high proportion of bilateral reflux, bilateral reflux caused damage to both sides, and there were fewer cases in which the partial renal function of one kidney decreased below 40%. The positive rates of ultrasound in antibiotic group and injection group were 45.5% (10/22) and 80.0% (24/30), respectively, and the difference was statistically significant (*P* = 0.010). The positive ultrasonographic findings were mainly renal collecting system dilatation, which may indicate high grade VUR clinically. Therefore, renal scar was found by renal isotope scan or renal collecting system was dilated by ultrasound in VUR children, we recommend for the indications for endoscopic injection. In 2017, the multivariate analysis showed that absent renal scars (*p* = 0.04) were statistically significant predictors of resolution [12].

Given these excellent resolution rates, nonoperative management with continuous antibiotic prophylaxis and imaging follow-up is a frequent choice for initial treatment. Guidelines in both the United States and Europe recommend infection prevention as initial treatment [2, 3], especially in the first year after diagnosis of VUR, when children with high-grade VUR are treated with antibiotics for infection prevention. Continuous low-dose antibiotic prophylactic treatment can prevent the recurrence of urinary tract infection and maintain the sterility of urine, thus preventing bacterial urinary reflux from causing kidney damage [2]. Therefore, the use of antibiotics to prevent infection is an important part of the treatment of VUR. The 2014 RIVUR trial report found that the use of antibiotics can reduce the recurrence rate of UTI by 50% [13], so many people believe that the reduction in recurrent urinary tract infections is a strong reason to advocate antibiotic treatment. However, there was no difference in kidney scarring between the treatment and placebo groups in the study (10.2% versus 11.9%). In 428 children, VCUG regurgitant regression was 50.9%, improvement 23.4%, unchanged 18.5%, and worse 7.2% [13]. Antibiotic prophylaxis did not have any benefit in reducing kidney scarring [14].

Antibiotic treatment also has disadvantages and negative effects. Antibiotic resistance is a major problem in the long-term use of antibiotic to prevent infections. The progression of antibiotic resistance in children with breakthrough urinary tract infections has been widely reported in the pediatric urology literature, but the role of the microbiome in this process remains uncertain [15, 16]. The degree of implementation of antibiotic therapy is also a troubling problem in clinical practice. Long-term adherence to daily prophylactic doses of antibiotics is questionable, and recent studies have shown that it is not 100% certain that patients are receiving the right dose to prevent infection [17]. In an earlier study, 97% of parents reported compliance with low-dose daily antibiotic medication to prevent infection, however, the drug was found in the urine of only 31% of patients [18]. Patient compliance is one of the biggest problems with antimicrobial prophylaxis.

Endoscopic injection treating VUR has become an alternative not only to open surgery, but also to conservative treatment, thus occupying an important position. Endoscopic surgery is a safe procedure with a low risk of complications and is currently the preferred method for most urologists and parents of children over 1 year of age [19, 20].The advantages of endoscopic injection surgery, which can usually be performed under general anesthesia for a short time (usually less than 20 min), are simple to operate, have minimal postoperative pain, and do not require postoperative catheter drainage. A meta-analysis of endoscopic injection outcomes showed that the success rates was 78.5% for grade I and II, 72% for Grade III, 63% for Grade IV and 51% for Grade V [6]. In our study, the single injection success rate was 57.4% (31/54), success rate of grade II reflux was 50%, grade III was 68.4%, grade IV was 55.0%, and grade V was 45.5%. After second injections, the overall success rate increased to 64.8% (35/54).

Injection surgery can be performed multiple times in the same child to increase the success rate. Ureteral replantation can also be performed after injection failure, without increasing the difficulty of the operation and affecting the effect of the operation. Puri [21] reviewed 149 cases of Deflux injection in which 19 cases completed three injections and no obstruction occurred. The clinical experience of the Queen Mary Hospital of the University of Hong Kong [22] showed that the success rate of single injection of Deflux under cystoscope for the treatment of Grade II-IV reflux was 100%, 64.5% and 80%, respectively. According to a meta-analysis conducted in 2010, in 5527 patients and 8101 ureteral units, VUR resolution after a single endoscopic treatment with Dx/HA was 78.5% for grade I and II, 72% for grade III, 63% for grade IV, and 51% for grade V [6]. The success rate of the second injection is 68% and the third is 34%. The overall success rate of injections was 85%. In our study, we attributed the slightly lower success rate of the injection to the immature technique during the early phase. Moreover, only two children received a second injection.

Several limitations of our study should be acknowledged. First, it is a retrospective review and selection bias might exist because the treatment dependent on surgeons and parental preference. Second, it does not collect information about Bladder and bowel dysfunction (BBD) in the study. Bladder and bowel dysfunction (BBD) is a known risk factor for febrile UTI with VUR. Finally, the sample size is small, which influenced the results to some extent. There is no further stratification for reflux grade and age with univariate and multivariate analysis. In the future, large-scale prospective study will be necessary to factors for treatment success.

Endoscopic injection is easy to operate with short surgical time and hospital stay, so it is a safe and feasible treatment. For the treatment of primary vesicoureteral reflux in children, the radiological resolution rate of endoscopic injection is better than antibiotic therapy. In this study, the positive expression of kidney scars on DMSA and the dilated of the collecting system on ultrasound are the indications for endoscopic injection.

## Data Availability

No datasets were generated or analysed during the current study.
